# Progress of equalizing basic public health services in Southwest China--- health education delivery in primary healthcare sectors

**DOI:** 10.1186/s12913-020-05120-w

**Published:** 2020-03-24

**Authors:** Rui Zhang, Yong Chen, Shili Liu, Shengxiang Liang, Geng Wang, Li Li, Xingneng Luo, Ying Li

**Affiliations:** 1 Department of Social Medicine and Health Service Management, Army Medical University (Third Military Medical University), No.30 Gaotanyan Road, Shapingba District, Chongqing, 400038 China; 2 Tuwai community health service center, Shapingba District, Chongqing, China

**Keywords:** Basic public health service, Health education, Mixed research methods, China

## Abstract

**Background:**

Equalizing basic public health services (BPHS) for all has been one goal of the health system reform in China since 2009. At the end of the 12th five-year plan, we conducted a series of surveys to understand BPHS implementation in Southwest China, and firstly reported implementation of health education (HE) and explore the barriers to HE delivery.

**Methods:**

Mixed research methods were used to investigate achievement in and barriers to HE in Southwest China. SPSS 22.0 was used for data analysis.

**Results:**

Nine hundred and eighty-nine residents were surveyed by questionnaire. 16 health care workers (HCWs) and 16 directors from 16 PHC sectors were included in the in-depth interviews. Less than 50% of residents who knew or utilized some item of HE. Age, residence, region (Chongqing or Guizhou), marital status, education, occupation, type and quality of primary health care (PHC) sectors to deliver BPHS, self-reported health and status of chronic diseases were associated with knowledge or utilization of HE. Distance to PHC sectors was associated with the knowledge of HE, gender and health insurance were associated with utilization of HE. Age, marital status, occupation region and self-reported health were associated with satisfaction regarding HE. Barriers to HE delivery included defects in HE design, weak capacity in PHC sectors to provide HE, residents’ poor cooperation, lack of multi-sector cooperation, poor equipment and weak health system.

**Conclusions:**

Southwest China delivered HE in all PHC sectors. However, our study underlined many barriers to equalization of HE. To address those barriers and achieve HE quality improvement, comprehensive measures to improve capacity of PHC sectors, enhance multi-sector cooperation and strengthen health information systems are all urgent needs.

## Background

Health equity is an essential human right and was defined as the pursuit of “striving for the highest possible standard of health for all people and giving special attention to the needs of those at greatest risk of poor health, based on social conditions” [1]. In recent years, health equity has risen to prominence on policy agendas in the wake of the universal health coverage movement [2–4] and landmark international reports on inequality in health [5, 6] and health care [7]. In China, health equity was emphasized in the Chinese new health care reform launched in 2009 [8]. One of the important strategies to promote health equity in this new health care reform is to promote universal access to public health services and for government to set the goal of equalizing access to basic public health services (BPHS) [9].

Equalized access to BPHS means residents in urban and rural areas can enjoy the equalization of BPHS freely [9]. Central government sets up the core basic package of BPHS, and local governments may add extended packages to it. Funding for BPHS is provided by government and increases year to year. In 2009, the core basic package of BPHS included nine categories (residents’ health records, health education, immunizations, health management for children, infectious disease prevention and control, maternal health management, health management for the aged, chronic disease management and management of patients with severe mental illness) and 21 items with 15CNY(Chinese Yuan) subsidies per-person were proposed [9, 10]. With social development, the government required enlarging the content of BPHS and gave more subsidies to the public [11–15]. In 2016, BPHS had 12 categories and 48 items (included two TB (Tuberculosis) management items compared with 2015) with 45 CNY subsidies per-person [15].

In China, primary health care (PHC) sectors, including community health centers (CHCs) and community health stations (CHSs) in urban areas, and township health centers (THCs) and village clinics in rural areas, play crucial roles in BPHS delivery [9]. China established PHC sectors in 1949, but undergone tremendous changes since the early 1980s and began to weaken [16–18]. Fortunately, China has re-emphasized PHC since the late 1990s [19] and intended to establish qualified PHC sectors in 2006 [20, 21]. Rebuilding the PHC system was given the central place in China’s health reform announced in 2009 [22, 23]. Several measures included an investment of CNY 850 billion (USD (United States dollar) 127 billion) to develop infrastructure and human resources for PHC sectors across the nation, and the operational cost of PHC were from governmental subsidies and service charges rather than relying on sales of drugs have been taken since 2009 [24–26]. PHC across the country has developed very fast recently. By the end of 2016, there were 926,518 PHC sectors, 34,327 CHCs, 36,795 THCs and 638,763 village clinics [27]. However, PHC development varied substantially in different economic area. CHCs in eastern China are better than in western China in infrastructure [28–30]. PHCs in western China are less developed and lack human resources [29–31].

BPHS has been carried out for 11 years, while national assessment of BPHS delivery in PHC sectors has only been done once in 2010 [32], which indicated that though BPHS have achieved some progress in equalization, also has confronted challenges. For example, the quality of BPHS was low; western China lags behind central and eastern China; and human resources in PHC sectors are the primary barriers to BPHS [32]. BPHS has also been carried out in western China since 2009. However, there is no systematic evaluation of BPHS delivery in this region. At the end of the 12^th^five-year plan (2015), we conducted a series of surveys to explore the achievements in and barriers to BPHS delivery in Southwest China, where PHC is less developed.

Southwest China is an underdeveloped area in China, whose per capita net income is much lower than that of Eastern and Central China [33]. Chongqing and Guizhou were the most important and representative provinces in Southwest China. BPHS had been carried out since 2009 in Chongqing and 2010 in Guizhou. PHC in Chongqing developed fast recently, almost all PHC sectors were held by the government [34], and had enough buildings [31]. However, all PHC sectors were short of qualified HCWs for BPHS (less than one public health professional per 100,000 people; more than 1/3 HCWs aged 45+; around 70% HCWs with only a college education or below and primary professional title or no title) [31]. PHC are less developed in Guizhou compared with Chongqing [29, 35]. Less than 20% of CHCs in urban areas were held and funded by the government. Almost 60% of CHCs had to rent buildings and only 31.5% of CHCs met the infrastructure requirements to provide PHC, only 58% of HCWs attained a secondary school education or below and 70% of HCWs had only a primary professional title or no title [29].

Since China’s central government set the first stage of BPHS in 2009, HE (health education), was helpful to disease control and health literacy [36], was one of the most important program [9]. HE, conducted by community health service center, is provided for all residents freely to disseminate health care knowledge, which included five items: provision of health education materials (PHEM), such as VCD and DVD, propagandizing columns of health education (PCHE), health counseling (HC) including health lifestyle counseling, health lectures (HL) for the health information, health behaviors and personalized health education (PHE) including home-guide for health lifestyle.

Therefore, in this study, we purposively selected Chongqing Municipality (as a region with more developed socio-economic development, with the Gross Domestic Product (GDP) was 2.04 trillion CNY, per capita GDP was 66.2 thousand CNY in Chongqing.) and Guizhou province (as a region with less developed socio-economic conditions, with 1.48 trillion CNY in Guizhou, per capita GDP was 41.4 thousand CNY in Guizhou) as study regions [37] to explore the achievements and barriers to HE delivery in Southwest China, then to better addressing the health disparities in China. In this study, we not only focused on assessing the delivery of HE program from the perspectives of both health care providers and users with structured questionnaires and in-depth interviews, but explored the specific barriers to HE delivery in PHC sectors from the health care providers to provide evidence for promotion of health equity.

## Methods

This cross-sectional survey utilized mixed research methods to collect data from August 2015 to January 2016. Quantitative research and qualitative research methods were used to assess delivery of HE in PHC sectors from community residents and HCWs, respectively.

A multi-stage randomized sampling was used and we selected one county/district to represent the socio-economic development of Guizhou and Chongqing respectively. PHC sectors in each selected county/district were divided into THCs in rural and CHCs in urban areas. THCs and CHCs were divided into developed and less developed groups for the sample representation and comprehensive understanding of the status of HMA. Finally, four THCs and four CHCs were random selected from each province, two THCs/CHCs that were more developed and another two less developed. Totally eight THCs and eight CHSs were chosen as final study regions. The flow chart of study region selection was shown in Additional file 3.

### Study participants and data collection

#### Quantitative research

We used consecutive sampling method to recruit participants. All people who showed up in the selected THCs/CHCs, met inclusion criteria and were interested in our study during our study period were recruited as participants. Exclusion criteria included: (1) those who had a mental illness or disturbed consciousness; (2) those who had difficulties with speech or hearing; (3) those who declined to participate in the survey. All participants had completed informed consent. A structural questionnaire with 4 sections was conducted to collect data, included socio-demographic information (age, gender, height, weight, education, residence, occupation before retirement, health insurance, and distance to PHC sectors), knowledge, utilization of and satisfaction with HE. This questionnaire was designed by our research team who reviewed the existing literature reports and then consulted related experts before pilot study. Then it was a pilot test with 100 participants. All questionnaires were executed by trained investigators from our research group and the completed questionnaires were checked and examined by trained investigators for quality control.

#### Qualitative research

**Fig. 1:**
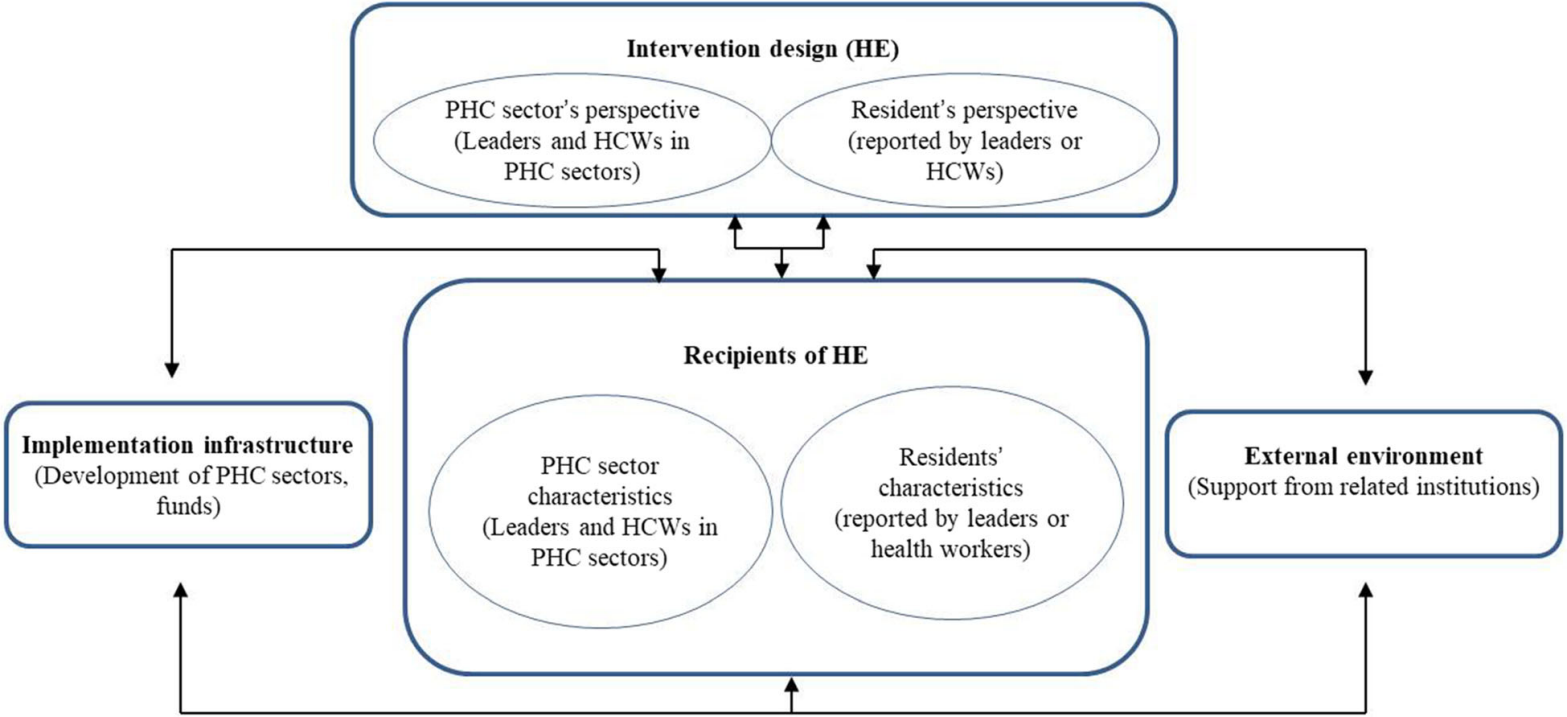
The Practical Robust Implementation and Sustainability Model (PRISM) for basic public health service. This figure presents the core domains of PRISM for basic public health service (BPHS). The interventions design is HE program design; the recipients include primary health care (PHC) sector and residents; the external environment is multi-sectors cooperation across related institutions; the organizational implementation and sustainability infrastructures include PHC sector’s infrastructure for HE and other essential infrastructures

In-depth interviews were used to explore barriers to delivery of HE from HCWs who provide HE to residents and leaders who are responsible for BPHS. 32 in-depth interviews with 16 LHWs and 16 leaders from the department of public health in PHC sectors enrolled in our study were purposively selected. A semi-structured topic guide was used for all interviews. The PRISM (Practical Robust Implementation and Sustainability Model) [38] widely used as a theoretical framework in Implementation Research [39, 40] to guide topic design. With the guide of PRISM, we collected data on barriers to HE delivery from the following aspects: HE design (interventions), PHC sectors’ characteristics and residents (recipients), cooperation across related institutions (external environment), and PHC infrastructure for HE (organizational implementation and sustainability infrastructure) (Fig. 1). All interviews were conducted in Mandarin in local meeting rooms. A senior researcher conducted all the interviews. Each interview lasted about 40–60 min. All interviews were audio-recorded with consent of participants and professionally transcribed for analysis.

### Data analysis

#### Quantitative analysis

Epi Data 3.1 was used to enter data. The data were analyzed using the Statistical Package for Social Science (SPSS 22.0). A two-tailed probability level of *p* < 0.05 was chosen as the level of statistical significance. Missing data were excluded from analysis. Descriptive statistics were used to describe study participants’ characteristics, knowledge about HE, utilization of HE and satisfaction with HE provision. Factors associated with knowledge, utilization and satisfaction screened by the Chi-square test (*p* < 0.05) were entered in multivariate logistic regression models (having no knowledge of HE =0, no use of HE =0, dissatisfaction with HE =0; having knowledge of HE =1, use of HE =1, satisfaction with HE = 1), which were used to examine the associations of those factors on knowledge, utilization and satisfaction.

#### Qualitative analysis

The framework approach [41, 42] was used to analyze all qualitative data following a five-step process: familiarizing, indexing each transcript with a framework, summarizing data in an analytical framework, data synthesis and interpretation of data [40, 43]. Following the framework approach, all interviews were transcribed into a Word document, and then all transcripts were coded and classified. We generated themes on barriers for each part of PRISM. The names of all participants in interviews were removed from the quotations in the results to preserve anonymity.

## Results

### Characteristics of participants

Totally 1004 community members were approached to participate; 15 declined to participate in the survey, and 989 completed the survey questionnaire (response rate was 98.5% (989/1004)). Among them, > 70%(724) were 60–80 years old and more than half (62.9%) were female; almost 60% were from urban areas and from Chongqing; 58.5 and 61.6% participants received HE from THCs and PHC sectors with better quality, respectively. The majority (77.3%) lived with a spouse. Among those participants, more than half had only a primary school education or no education and were peasants. Basic health insurance covered almost all of those residents. Close to 80% lived close to PHC sectors (less than 1 km). Notably, close to 30% reported poor health and more than 70% had chronic diseases (Table 1).

**Table 1:** Demographic characteristics of the questionnaire respondents

Characteristics	Number	Percentage
**Age(*n*** **= 989)**		
<50	77	7.8
50–60	131	13.2
60–70	449	45.4
70–80	275	27.8
≥ 80	57	5.8
**Gender(*n*** **= 988)**		
Male	367	37.1
Female	621	62.9
**Residence (*n*** **= 982)**		
Rural	406	41.3
Urban	576	58.7
**Region (*n*** **= 989)**		
Chongqing	586	59.3
Guizhou	403	40.7
**Type of PHCs(*n*** **= 989)**		
Township hospitals	579	58.5
Community health centers	410	41.5
**Quality of health service in PHCs(*n*** **= 989)**		
Good	609	61.6
Poor	380	38.4
**Marital status(*n*** **= 984)**		
Married	761	77.3
Divorced / Widowed	223	22.7
**Education (*n*** **= 988)**		
Primary and below	562	56.9
Middle school	266	26.9
College and above	160	16.2
**Occupation (*n*** **= 981)**		
Employed in enterprises/institutions/government	355	36.2
Peasants/ rural migrant workers	555	56.6
Others	71	7.2
**Health insurance(*n*** **= 988)**		
Basic health insurance	965	97.7
Others	23	2.3
**Distance to PHC (*n*** **= 983)**		
< 1 km	782	79.6
1-2 km	112	11.4
≥ 2 km	89	9.1
**Self-reported health(*n*** **= 987)**		
Well	319	32.3
Fair	378	38.3
Unwell	290	29.4
**With Chronic diseases (*n*** **= 989)**		
Yes	760	76.8
No	229	23.2

16 HCWs delivering HE were interviewed. Most of them were nurses before delivering BPHS. Almost all (14/16) HCWs for HE undertook more than one item of BPHS. Half had delivered BPHS for less than two years, and more than 1/3 for less than one year. Most of them had only a junior professional title or no professional title. Most of them were < 35 years old. Sixteen directors in the department of BPHS of 16 PHC sectors were included in in-depth interviews as well.

### Knowledge, utilization of and satisfaction with HE among residents and associated factors

**Fig. 2:**
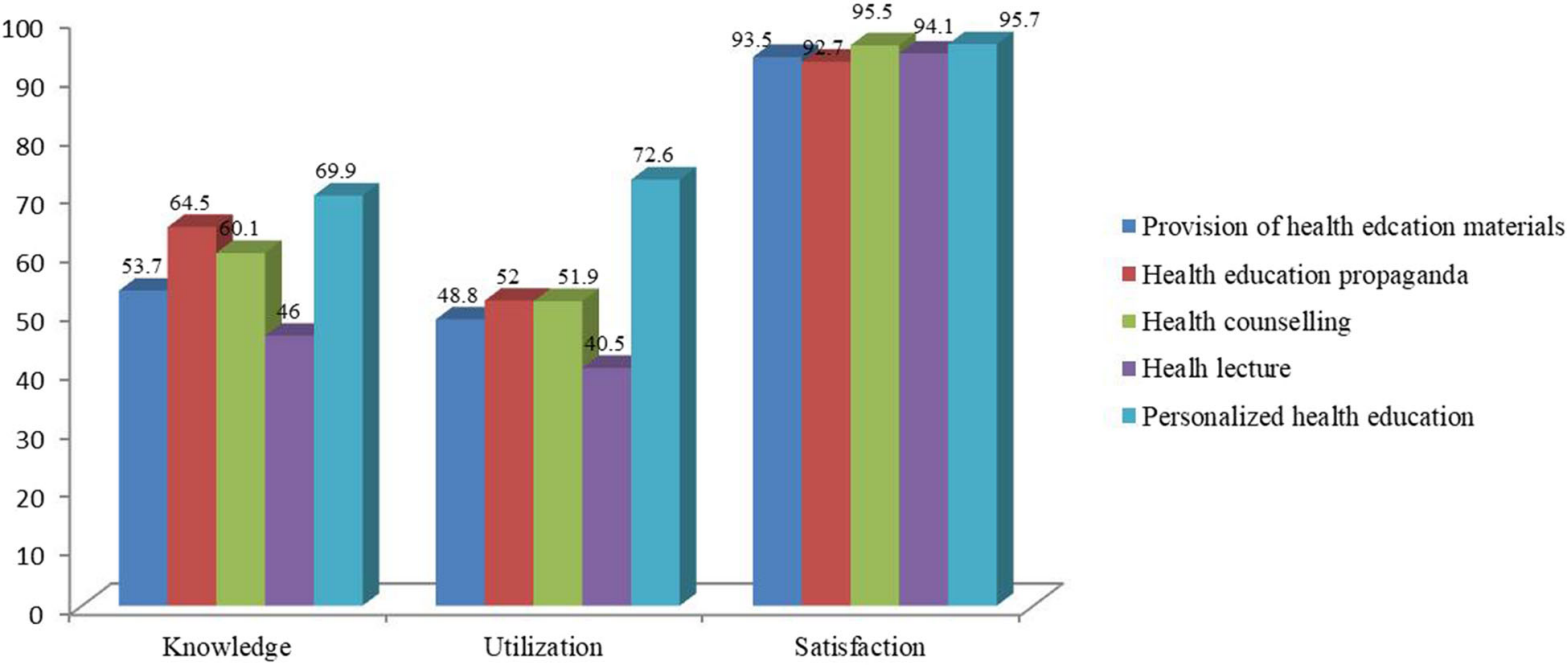
Knowledge and utilization of, and satisfaction to HE. This figure presents the percentage of residents had knowledge and utilization of the programs of health education (HE), the percentage of residents satisfied to the programs of health education (HE)

Less than 50% (445) had knowledge about provision of HL in PHCs, and around 50% (520) knew PHEM in PHCs (Fig. 2). Results regarding utilization of HE were similar to the results for knowledge, and all items of HE except for PHE were used by less than 60% of residents. However, more than 90% of residents were satisfied with HE (Fig. 2).

We also explored factors associated with knowledge and utilization of, and satisfaction with HE among residents (Tables 2, 3 and 4). As for factors associated with knowledge about HE, multivariate logistic regression showed that the elderly had less knowledge about PHEM and were less likely to use PCHE; residents from urban areas had more knowledge of all items of HE except for PCHE, and were more likely to use PHEM and PCHE; residents from the Guizhou had less knowledge of and were less likely to use all items of HE; residents received HE from CHCs had less knowledge of and were less likely to use PHEM; residents who received HE from PHCs of poor quality had less knowledge of three items of HE included PHEM, PCHE and HL, and use HL less; divorced/widowed residents had less knowledge of PCHE and had used PHEM and PCHE less; residents with higher education had better knowledge of PCHE; for residents with a middle-school education, more had used PCHE; for residents with education of college and above, more used PHEM, PCHE and HC; Residents who were peasants/rural migrant workers and other workers know less of PCHE, and were less likely to use PHE; residents who lived a moderate distance (with 1-2 km to PHCs) from PHC sectors had less knowledge of PHEM; residents whose self-reported health was “fair” had less knowledge about PHE and were less likely to use HC and PHE; residents with “unwell” health status had used HC and HL less; residents without any chronic diseases had less knowledge of three items of HE (PHEM, HL and PHE), and less use of HL. In addition, female residents were less likely to use PCHE and residents without basic health insurance were more likely to use HL.

**Table 2:** Multivariate analysis for factors associated with resident’s knowledge about Health education

Variable	HE				
	PHEM	PCHE	HC	HL	PHE
**The elderly(*n*** **= 989)**					
No	1	–	–	–	–
Yes	0.64(0.44,0.94)	–	–	–	–
**Marital status(*n*** **= 984)**					
Married	1	1	–	–	–
Divorced / Widowed	0.73(0.53,1.01)	0.68(0.48,0.95)	–	–	–
**Education (*n*** **= 988)**					
Primary and below	–	1	–	–	–
Middle school	–	1.69(1.17,2.46)	–	–	–
College and above	–	3.27(1.94,5.51)	–	–	–
**Occupation (*n*** **= 981)**					
Employed in enterprises/institutions/government	–	1	–	–	–
Peasants/ rural migrant workers	–	0.54(0.37,0.80)	–	–	–
Others	–	0.40(0.22,0.71)	–	–	–
**Residence(*n*** **= 982)**					
Rural	1	–	1	1	1
Urban	2.26(1.59,3.23)	–	1.59(1.21,2.10)	1.67(1.26,2.22)	1.74(1.31,2.33)
**Region (*n*** **= 989)**					
Chongqing	1	1	1	1	1
Guizhou	0.48(0.33,0.70)	0.29(0.21,0.41)	0.30(0.23,0.39)	0.36(0.26,0.48)	0.50(0.38,0.67)
**Type of PHCs(*n*** **= 989)**					
Township hospitals	1	–	–	–	–
Community health centers	0.68(0.47,0.98)	–	–	–	–
**Quality of health service in PHCs**					
Good	1	1	–	1	–
Poor	0.60(0.43,0.84)	0.50(0.36,0.68)	–	0.48(0.35,0.66)	–
**Distance to PHC (*n*** **= 973)**					
<1 km	1	–	–	–	–
1-2 km	0.55(0.36,0.85)	–	–	–	–
≥ 2 km	0.87(0.54,1.41)	–	–	–	–
**Self-reported health(*n*** **= 987)**					
Well	–	–	–	–	1
Fair	–	–	–	–	0.55(0.39,0.77)
Unwell	–	–	–	–	0.74(0.50,1.08)
**With Chronic diseases (*n*** **= 989)**					
Yes	1	–	–	1	1
No	0.64(0.44,0.94)	–	–	0.59(0.43,0.82)	0.70(0.50,0.98)

Notes: *HE* refers to health education, *PHEM* refers to Provision of health education materials, *PCHE* refers to Propagandizing column of health education, *HC* refers to Health counseling, *HL* refers to Health lecture, *PHE* refers to personalized health education, −-refers to variables excluded in the model

**Table 3:** Multivariate analysis for factors associated with resident’s utilization of Health education

Variable	HE				
	PHEM	PCHE	HC	HL	PHE
**The elderly(*n*** **= 989)**					
No	–	1	–	–	–
Yes	–	0.65(0.43,0.98)	–	–	–
**Gender (*n*** **= 989)**					
Male	–	1	–	–	–
Female	–	0.66(0.48,0.91)	–	–	–
**Marital status(*n*** **= 984)**					
Married	1	1	–	–	–
Divorced / Widowed	0.63(0.44,0.92)	0.61(0.42,0.90)	–	–	–
**Education (*n*** **= 988)**					
Primary and below	1	1	1	–	–
Middle school	1.41(0.97,2.05)	1.45(1.01,2.08)	0.84(0.58,1.21)	–	–
College and above	2.22(1.37,3.59)	2.52(1.57,4.05)	1.62(1.02,2.58)	–	–
**Residence(*n*** **= 982)**					
Rural	1	1	1	–	–
Urban	1.61(1.13,2.30)	2.13(1.52,2.98)	1.40(1.00,1.95)	–	–
**Occupation (*n*** **= 981)**					
Employed in enterprises/institutions/government	–	–	–	–	1
Peasants/ rural migrant workers	–	–	–	–	0.43(0.30,0.63)
Others	–	–	–	–	0.34(0.19,0.62)
**Region (*n*** **= 989)**					
Chongqing	1	1	1	1	1
Guizhou	0.36(0.26,0.50)	0.28(0.20,0.40)	0.17(0.13,0.24)	0.16(0.12,0.23)	0.18(0.13,0.26)
**Type of PHCs(*n*** **= 989)**					
THCs	1	–	–	–	–
CHCs	0.66(0.44,0.99)	–	–	–	–
**Quality of health service in PHCs**					
Good	–	–	–	1	–
Poor	–	–	–	0.52(0.36,0.76)	–
**Health insurance(*n*** **= 988)**					
Basic health insurance	–	–	–	1	–
Others	–	–	–	3.04(1.17,9.91)	–
**Self reported health(*n*** **= 987)**					
Well	–	–	1	1	1
Fair	–	–	0.67(0.47,0.97)	1.11(0.76,1.62)	0.53(0.36,0.79)
Unwell	–	–	0.62(0.42,0.90)	0.64(0.42,0.97)	0.77(0.50,1.18)
**With Chronic diseases (*n*** **= 989)**					
Yes	–	–	–	1	–
No	–	–	–	0.67(0.45,0.99)	–

Notes: *HE* refers to health education, *PHEM* refers to Provision of health education materials, *PCHE* refers to Propagandizing column of health education, *HC* refers to Health counseling, *HL* refers to Health lecture, *PHE* refers to personalized health education, −-refers to variables excluded in the model

**Table 4:** Multivariate analysis for factors associated with resident’s satisfaction with Health education

Variable	HE				
	PHEM	PCHE	HC	HL	PHE
**The elderly(*n*** **= 989)**					
No	1	–	1	–	1
Yes	3.14(1.26,7.85)	–	3.28(1.21,8.90)	–	2.80(1.17,6.69)
**Marital status(*n*** **= 984)**					
Married	–	–	–	–	1
Divorced / Widowed	–	–	–	–	0.33(0.14,0.78)
**Occupation (*n*** **= 981)**					
Employed in enterprises/institutions/government	1	–	–	–	–
Peasants/ rural migrant workers	0.50(0.18,1.41)	–	–	–	–
Others	0.19(0.05,0.77)	–	–	–	–
**Region (*n*** **= 989)**					
Chongqing	–	1	–	1	–
Guizhou	–	0.39(0.17,0.90)	–	0.27(0.10,0.72)	–
**Self reported health(*n*** **= 987)**					
Well	–	1	–	–	–
Fair	–	0.24(0.07,0.85)	–	–	–
Unwell	–	0.25(0.07,0.95)	–	–	–

Notes: *HE* refers to health education, *PHEM* refers to Provision of health education materials, *PCHE* refers to Propagandizing column of health education, *HC* refers to Health counseling, *HL* refers to Health lecture, *PHE* refers to personalized health education, −-refers to variables excluded in the model

Regarding factors associated with satisfaction with HE, multivariate logistic regression revealed that the elderly were more likely to be satisfied with three items of HE, included PHEM, HC and PHE. Residents in Guizhou were less likely to be satisfied with PCHE and HL; less satisfaction with PHE was observed among divorced/widowed residents; Peasants/ rural migrant workers and other workers were less satisfied with PHEM; residents with self-reported worse health status were less satisfied with PCHE.

### Barriers to HE delivery in PHC sectors

All HPC sectors believed that HE programs benefited residents. But HCWs and leaders in in-depth interviews reported numerous barriers to provision of HE.

Barriers to HE implementation were reported from the four core PRISM domains (Table 5):

1. ***Interventions:*** On one hand, PHC sectors lacked sufficient materials for HE. On the other hand, the current materials do not meet the health needs of local residents in content and format, particularly the elderly with poor hearing, poor memory and low education. In addition, PHC sectors often offered gifts to attract residents to participate in HE activities, but such gifts were not covered by the funds from BPHS.
2. ***Recipients*:** All PHC sectors were short of full-time professional HCWs for HE, and HCWs had difficulty providing HC and PHE. The majority of HCPs were neither satisfied with their salary nor with opportunities for self-development. Some complained that the lower authority of PHC sectors among residents influenced their participation in HE activities. Most HCWs reported that residents had no correct recognition of HE activities and were therefore reluctant to participate in HL, utilize HC, or get and read the HE materials. Some residents were not able to understand HE content due to lower education, particularly the elderly.
3. ***External environment:*** Some HCWs disclosed that it is difficult to provide the appropriate venue for health lectures where video materials on health knowledge can be shown, and which is accessible for residents, as there is often only a small meeting room in PHCs. HCWs also complained that cooperation of multi-sectors was not so good.
4. ***Implementation infrastructure:*** Lack of transportation modes in PHC sectors to carry out health education in remote mountainous areas were reported by some HCWs.

**Table 5:** Barriers in implementation of HE

Core PRISM domains	Themes	Results	Example quotations
**Interventions –HE program design**	Content/materials for HE	The materials for HE cannot meet needs: The materials for HE provided by CDC cannot meet needs of HE, and so HCWs in PHC have to look for the content on health issues for each topic. They were not sure the credibility of the content identified by them based on their knowledge. On the other hand, they felt difficult to find enough content because they are required to change the content every two months. HCWs lacked of hard copy and video materials to hand out for residents when they carry out “Provision of health education materials”. Content of lecture could not meet needs of residents too.	We have difficulty in HL. Our CHC is not teaching hospital, we had not many teaching PPT for HL. Though CDC often give us some materials for HL, it is not enough. We just get some materials for HL from websites, or prepared the PPT by ourselves. But those materials were limited and are not enough for 12 HL per year. And so the residents would not like to participate in our HL when we repeated HL. This is a big difficulty *(HCWs in CHC).* Some materials for HE are not available in our PHC sector and we were required to look for by ourselves. But we had no adequate knowledge to prepare the materials based on the needs of residents. CDC often gave some materials for some health issues, but not all of these materials were needed by residents because they were not based on resident’s health needs *(HCWs in CHC)*.
	Funds	Almost all HCWs mentioned lack of enough funds for HE materials preparation and activities because PHC sectors needed prepare gifts for participants which were not covered by funds from BPHS	Actually, the funds are not enough. We paid for materials for HE by our CHC. And we often bought gifts for residents in order to attract them to participate in HE activities. *(HCWs in THC).*
**Recipients**	PHC sectors	PHCs lack of professional HCWs for HE: Almost all HCWs reported they did not work on HE full-time and undertake more than one item of BPHS and they lacked of health knowledge. Particularly HCWs reported they had little skills to provide health counseling and personalized health education. Majorities of interviewers were not satisfied with their salary and the opportunity of self-development. Lower authority of PHC among residents results low participation of residents in HE activities	The biggest difficulty is lack of professional HCWs for HE. We are part-time working for HE *(HCWs in CHC).*. We felt difficult to provide PHE for residents which required providers with highly professional knowledge. We are not GP who has knowledge of both internal medicine and surgery. So we cannot provide PHE of high quality. We lacked of human resource *(HCWs in CHC).* We lacked of knowledge of public health, we are nurses. We don’t know lots of professional health knowledge *(HCWs in CHC).*. Our salary is very low. I am Contract worker, income is very low *(HCWs in THC).*. My major is Family planning and I cannot see good prospect of myself development *(HCWs in CHC).*
	Residents	Residents had no correct recognition of HE activities and would not like to participate in HE: most of HCWs reported that residents were reluctant to participate in HL, utilize HC, get and read the materials because residents had no correct recognition of HE or they cannot discern the actual HE from advertisement by drug dealers. PHCs often used gifts to attract residents to participate in HL. Some HCWs reported some residents cannot understand HE content due to lower education, particularly the elderly.	If we invite many doctors to join our HE activities and give gifts to residents, residents would like to participate in our activities, and otherwise they have no interesting in HE activities *(HCWs in THC).* They (residents) thought we are drug dealers to give advertisement to sell medicine. They would not like to participate. Some residents thought it waste time to listen half hour lectures which is not addressing their health problems *(HCWs in CHC).* We had PCHE in our THC, but many residents don’t know it is PCHE or never pay attention to it. Because most of the residents who use health service in THC were the elderly *(HCWs in THC).*
**External environment**	Venue for HE	Lack appropriate venue for health lectures It is difficult to have the venue where is appropriate for health lectures and playing video materials on health knowledge and are accessible for residents though there is a small meeting room in PHCs	We often have difficult to find an appropriate venue for HE activities outside of our CHC *(HCWs in CHC)*.
	Multi-sector cooperation	Several HCWs also complained multi-sectors cooperation was not so good. For example, the city management personnel often prohibited HCWs from having a site for HC or PHEM in the street because they thought those activities had impact on clean and tidy of street.	HE needs cooperation of multi-sectors. It is difficult to carry out HE activities only by CHS. We need organize residents, we need look for venue, and we need prepare HE materials for residents *(HCWs in THC).* It is difficult to have a venue for HE activities, the city management is strict. We want to have a banner for HC (in order to attract residents to come) in street, but the city management personnel would prohibit *(HCWs in CHC).*
**Implementation infrastructure**	Transportation tools	Some HCWs complained they lack of transportation tools to carry out health education in remote mountain area in many PHC sectors	We have no transportation tools for HE. We carry out HE activities in remote rural mountain area, but is not convenient, we had no car t to take materials *(HCWs in CHC)*.

Notes: *HE* refers to health education, *PHC* refers to primary health care, *HCWs* refers to health care workers, *HL* refers to health lectures, *PHE* refers to personalized health education, *HC* refers to health counseling, *PCHE* refers to propagandizing column of health education

## Discussion

HE delivery in PHC achieved substantial progress over the past eight years, since equalization of BPHS became one important goal of Chinese health reform. However, HE delivery still varied substantially among different regions in China during 2012–2016. Several studies reported knowledge, use of and satisfaction with HE during 2013–2016. More than 90% of residents used HE in the Shandong province during 2013–2014 [44] and 70% residents had knowledge of two items of HE (PCHE and PHEM) in the Liaoning province in 2014 [45]. In most provinces/regions, around 60% of residents had knowledge or use of some items of HE [45–48]. However, fewer than 50% of residents had knowledge of HE in the Hubei province in 2015 [48] and used HE in the Guangdong province in 2013 [49]. Notably, only 4.4% of residents participated in HL in 2014 in the Xinjiang province [50]. A study in the Hubei province reported satisfaction with HE, which disclosed that only 47.2% residents were satisfied with HE in 2015 [49]. Our study also found that fewer residents knew about HE materials, and only about 50% of residents knew about health lecture and health materials provisions in PHC sectors.

Implementation of HE varied among different populations in China. Firstly, Xu SY et al. [51] indicated that residents in rural were more likely to know HE in Anhui. Zhang H et al. [52] reported that no differences were observed on the utilization of HE between rural and urban areas in Shandong. Previous studies reported that residents in rural areas were more likely to use HE in the Shandong province during 2013–2014 [44]. While, our study observed that residents in urban areas had more knowledge on HE, and were more likely to use HE (PHEM, PCHE and HC). Secondly, previous studies indicated that age, education, gender, chronic disease status, occupation, health insurance, were associated with establishment and implementation of HE [51–54]. Our study similarly observed that knowledge, use of and satisfaction with HE varied significantly among residents of different ages, gender, education, occupation, marital and chronic disease status, quality of PHCs or health status.

### Barriers to equalizing basic public health in Southwest China

The previous studies [47, 55, 56] found that PHC sectors in China still face many problems and challenges in equalization of BPHS, despite all PHC sectors having carried out BPHS and made a lot of improvement. Our study consistently disclosed barriers to delivering HE in Southwest China.

Our study disclosed some problems in HE program design. Firstly, equalization of BPHS emphasizes providing BPHS in response to residents’ needs rather than providing the same BPHS to everybody [9]. National guidelines for BPHS also stated that HE requires a health needs assessment firstly [9]. However, we found that no comprehensive community health needs assessment existed; therefore, the content of HE and its approach could not be responsive to residents’ health needs, and residents therefore had no interest in receiving HE activities. Other studies also reported that the BPHS programs package did not adapt to actual health needs of local residents, because there was no scientific community needs assessment [47, 56]. A study in Kunming similarly reported that materials for HE were technical and not easily understood by residents [56]. Secondly, a study by Ding Y et al. reported that the subsidy of village doctors was not sufficient remuneration for their efforts to provide public health service [57]. Though the subsidies increased up to 45 CNY in 2016, we found that the subsidies were not enough to meet the increased needs of residents and to be incentives driving HCWs to provided qualified HE.

Building and strengthening PHC in China is one effective way to address health inequity. Although the Chinese government has paid great attention to the development of PHC sectors since 1997 [9–12, 58],inadequate numbers and insufficient competency of HCWs resulted in relatively heavy work load for HCWs and affected HE delivery [32, 33, 36, 55, 59, 60]. Previous studies have revealed that HCWs who deliver BPHS services were part-time workers, and consisted of many community nurses and only a few public health specialists [32, 46, 55, 59]. Studies also reported that most THCs lack professional HCWs for HE, which affected the implementation of HE [61–63]. PHC in western China were underdeveloped and did not meet the standardized requirement and lacked equipment, HCWs and funding [29, 35], therefore, PHC sectors in Western China had not yet prepared well for BPHS. Primarily, a shortage of enough qualified HCWs in PHCs was the main barrier. We found that heavy workloads, poor working conditions, low income, and a lack of social security were possible reasons for a failure to attract and retain qualified HCWs for HE in PHC sectors, which was consistent with previous study [60]. Furthermore, equipment in PHC sectors, such as essential infrastructure for BPHS implementation, has had an impact on BPHS. The PHC sectors with simple and crude conditions and equipment have difficulties attracting residents to participate in BPHS [63]. We further indicated that lack of appropriate venue for health lectures affected the implementation of HE. Our study, together with previous studies, observed that poor cooperation of residents resulted in less participation in HE, which is one of the main barriers to providing HE [33, 60, 63, 64], and the reasons for poor cooperation included incorrect understanding of HE, low health literacy [65, 66], low education, poor health awareness and a weak ability to understand health knowledge among residents, particularly in rural areas [61–64]. Another intervention study by Wen et al. proved that increased use of HE was observed among the elderly with improved health literacy [67]. HE can improve health literacy, including improving knowledge and developing life skills which are conducive to individual and community health [68]. Regarding BPHS, HE should be a priority in PHC sectors because high quality HE may improve health literacy of residents, promote recognition of all other programs of BPHS packages, and can assist residents in learning about PHC sectors and HCWs.

“Disease-based curative models” are deeply rooted in society, and health issues are more often managed in a disconnected and fragmented manner and coordination is frequently lacking across care providers, settings and time [69]. It is the same in BPHS implementation in China, and the concept of integrated care is lacking. Multi-sector cooperation as the external environment for HE implementation is not supportive. The previous study reported that BPHS cannot receive support from multi-sectors for ineffective cooperation and communication between PHC sectors, Community Committees, Sub-district Administration Offices and Health and Family Planning Commissions [47]**.** One study found that less than 10% of residents got knowledge of BPHS from PHC sectors in the Xinjiang province [50], which further proved that multi-sector cooperation is very important for residents to learn about BPHS. Without support from Community Committees to organize residents to participate in HE, it is very difficult for PHCWs to carry out HE [47]; without multi-sector cooperation, residents often misunderstand HE as the fraudulent ads on drugs and they were unwilling to participate in HE activities [47, 63, 64, 70]. Our study revealed PHC sectors often worked independently to educate and organize residents for the lack of cooperation across PHC sectors, grassroots government (Sub-district Administration Offices and Community Committees), security sectors (urban management offices) and employers; therefore, many residents could not discern HE services from drug sales.

## Strengths and limitations

Some previous studies focus on the effect of HE [71], some studies report on the situation of HE as a summary of the experiences of individual PHC sectors [61–63]. We used a combination of quantitative research methods and qualitative research methods to assess HE delivery from the perspectives of care providers and health-care consumers to maximize trustworthiness and credibility of data. We also aimed to provide sufficient in-depth descriptions of barriers to deliver HE from care providers and leaders in PHCs. However, we did not include policy-makers from local Health and Family Planning Commissions as study participants, who could have provided information about difficulty and suggestions to deal with barriers faced by PHCs. The questionnaire participants were recruited from PHC sectors, rather than from communities. Those residents may be more likely to trust PHC sectors and therefore have greater access to HE services than those who did not use health services in PHC sectors. So the rates of knowledge, utilization and satisfaction of community residents were likely lower than what we reports.

## Implications

Though the findings from our study may not generalize to other provinces in central and eastern China, where socio-economy and PHC sectors developed better compared to Southwest China., the results may have the following implications for regions in Western China or countries with the same socio-economic characteristics. Firstly, Residents and communities should be placed at the center of BPHS program design and HE planning. Training HCWs in PHC sectors to conduct community health needs assessments should be a primary concern. Secondly, residents’ health literacy, and their capacity to use HE and self-care will be improved by health education. Effective HE for residents should be further enlarged and strengthened. Research and training on how to carry out effective HE by HCWs in PHC sectors deserves consideration. Thirdly, development of HCWs’ competency in PHC sectors should be at the center of capacity building. Further studies on core competencies of HCWs to fill their roles in BPHS, education or training programs geared towards building those competencies should be encouraged and funded. Some strategies and incentives are essential to attract qualified HCWs to work in PHCs, such as introduction of a pension program, support of professional promotion, and bonuses are also important considerations as incentives [70]. Capacity building of PHC should also include PHC sectors’ infrastructure construction in Western China. Finally, strengthening multi-sector collaboration between health and non-health sectors, government and the private sector, and the collaboration between families, governments and the private sector would be a great support for HE.

## Conclusions

HE may be an important means to improve literacy and use of BPHS for residents. HE has been implemented widely in Southwest China, but was unbalanced among different populations. Many barriers in HE implementation were identified and there is no simple solution to the barriers. Apart from the funding, comprehensive measures to improve the capacity of PHC sectors, including HCWs’ competency and infrastructure, multi-sector cooperation and health information systems were potential solutions to address those barriers to improve the quality of HE, and finally lead to overall equalization of BPHS.

## Supplementary information

**Additional file 1.** STROBE Checklist. A checklist for the items that should be included in reports of cross-sectional studies

**Additional file 2.** Questionnaire. The questionnaire with an English version for this survey.

**Additional file 3.** Study design. The flow chart of study region selection.

## Data Availability

Data sharing is not applicable to this article as no datasets were generated or analyzed during the study.

## Abbreviations

BPHS: Basic Public Health Services
CHCs: Community Health Centers
CHSs: Community Health Stations
CNY: Chinese Yuan
HC: Health Counseling
HCWs: Health Care Workers
HE: Health Education
HL: Health Lectures
PCHE: Propagandizing Columns Of Health Education
PHC: Primary Health Care
PHE: Personalized Health Education
PHEM: Provision Of Health Education Materials
PRISM: Practical Robust Implementation and Sustainability Model
TB: Tuberculosis
THCs: Township Health Centers
USD: United States dollar
