# Supplementary material for: Progress of equalizing basic public health services in Southwest China--- health education delivery in primary healthcare sectors
Source: BMC Health Serv Res. 2020 Mar 24;20:247. doi: 10.1186/s12913-020-05120-w (PMC7092608; doi:10.1186/s12913-020-05120-w)
Supplement: Supplementary file 2 — Additional file 2. Questionnaire. The questionnaire with an English version for this survey. [file 12913_2020_5120_MOESM2_ESM.doc]

**Number：**

|  |  |  |  |
| --- | --- | --- | --- |

**Investigation of Progress of Equalizing basic public health services in Southwest China--- Health Education Delivery in Primary Healthcare Sectors**

**Instructions：**

Multiple-choice questions: Please tick the option based on your information, such as: "√a. male, b. female";

Fill in the blank question: Please fill in the number or text directly on "", such as "There are 3 people in your family now."

Table: Please fill in the answer in the right half of the table, do not directly mark the problem.

**Name：**

**Phone number：**

**Primary Health sectors：**

Investigator： Date of investigation： Inspectors： Date of inspect：

1. **Your gender：** a. Male b. Female
2. **Your age：**
3. **Your residence：** a. Rural b. Urban
4. **Where are you from?** Chongqing b. Guizhou
5. **Height：** m； weight： kg
6. **Your married status：** a. Unmarried b. Married c. Divorced d. Widowed
7. **Your education level：** a. Illiteracy b. Primary school c. Junior high school d. Senior high school e. College and above
8. **Your occupation**：a. Employed in enterprises/institutions b. Employed in government c. Peasants d. rural migrant workers e. students f. Others
9. **Your health insurance：**1）Basic health insurance 2）Others
10. **Your distance to Primary health care sectors**： KM
11. **Your self-reported health status？**

a. Well b. Fair c. Unwell

1. **Are you have chronic disease(hypertension, diabetics, coronary heart disease et al)**

a. Yes b. No

**13.The knowledge about, utilization of and satisfaction with Health education(HE)**

| Items of HMA | Do you know the HE service? | | Do you utilize the HE service? | | Do you satisfy with these HE service? | |
| --- | --- | --- | --- | --- | --- | --- |
|  | Yes | No | Yes | No | Yes | No |
| Provision of health materials(PHEM) |  |  |  |  |  |  |
| Propagandizing columns of health education (PCHE) |  |  |  |  |  |  |
| Health counselling (HC) |  |  |  |  |  |  |
| Health lectures (HL) |  |  |  |  |  |  |
| Personlised health education (PHE) |  |  |  |  |  |  |
