# Supplementary figures and images for: Progress of equalizing basic public health services in Southwest China--- health education delivery in primary healthcare sectors
Source: BMC Health Serv Res. 2020 Mar 24;20:247. doi: 10.1186/s12913-020-05120-w (PMC7092608; doi:10.1186/s12913-020-05120-w)

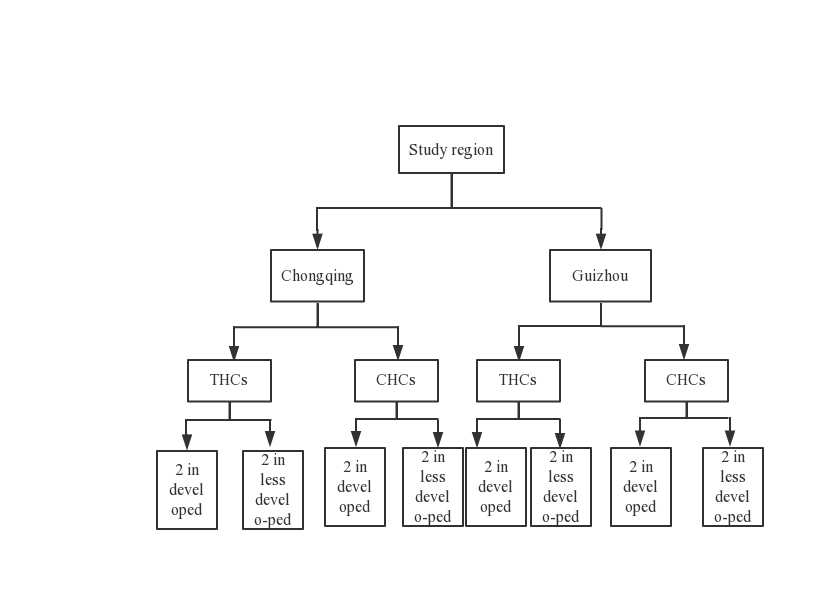

Supplement: Supplementary file 3 — Additional file 3. Study design. The flow chart of study region selection. [file 12913_2020_5120_MOESM3_ESM.jpg]
